# Registered nurses’ exposure to workplace aggression in Norway: 12-month prevalence rates, perpetrators, and current turnover intention

**DOI:** 10.1186/s12913-023-10306-z

**Published:** 2023-11-16

**Authors:** Solveig Osborg Ose, Signe Lohmann-Lafrenz, Silje L. Kaspersen, Hanne Berthelsen, Gunn Hege Marchand

**Affiliations:** 1 Health Services Research Group, SINTEF, Trondheim, Norway; 2 St. Olav’s University Hospital, Trondheim, Norway; 3 Centre for WorkLife and Evaluation Studies, Malmö University, Malmö, Sweden

**Keywords:** Workplace aggression, Physical Violence, Threats of Violence, Sexual Harassment, Bullying, Intention to leave, COPSOQ III, Occupational Health and Safety

## Abstract

**Background:**

Identifying occupational health hazards among Registered Nurses (RNs) and other health personnel and implementing effective preventive measures are crucial to the long-term sustainability of health services. The objectives of this study were (1) to assess the 12-month prevalence rates of exposure to workplace aggression, including physical violence, threats of violence, sexual harassment, and bullying; (2) to identify whether the perpetrators were colleagues, managers, subordinates, or patients and their relatives; (3) to determine whether previous exposure to these hazards was associated with RNs’ current turnover intention; and (4) to frame workplace aggression from an occupational health and safety perspective.

**Methods:**

The third version of the Copenhagen Psychosocial Questionnaire (COPSOQ III) was used to assess RNs’ exposure to workplace aggression and turnover intention. A national sample of 8,800 RNs in Norway, representative of the entire population of registered nurses in terms of gender and geography, was analysed. Binary and ordinal logistic regression analyses were conducted, and odds for exposure and intention to leave are presented, with and without controls for RNs’ gender, age, and the type of health service they work in.

**Results:**

The 12-month prevalence rates for exposure were 17.0% for physical violence, 32.5% for threats of violence, 12.6% for sexual harassment, and 10.5% for bullying. In total, 42.6% of the RNs had experienced at least one of these types of exposure during the past 12 months, and exposure to more than one of these hazards was common. Most perpetrators who committed physical acts and sexual harassment were patients, while bullying was usually committed by colleagues. There was a strong statistical association between exposure to all types of workplace aggression and RNs’ intention to leave. The strongest association was for bullying, which greatly increased the odds of looking for work elsewhere.

**Conclusions:**

Efforts to prevent exposure to workplace aggression should be emphasised to retain health personnel and to secure the supply of skilled healthcare workers. The results indicate a need for improvements. To ensure the sustainability of health services, labour and health authorities should join forces to develop effective workplace measures to strengthen prevention, mitigation, and preparedness regarding incidents of workplace aggression in health services and the response and recovery regarding incidents that could not be prevented.

**Supplementary Information:**

The online version contains supplementary material available at 10.1186/s12913-023-10306-z.

## Introduction

Workplace aggression is defined as behaviour that is intended to harm or intimidate employees and includes physical violence, verbal threats of violence, bullying, and harassment [1]. According to occupational health and safety (OHS) regulations and international labour standards, employers are obligated to take steps to address and prevent workplace aggression and to create a healthy and safe working environment [2]. Potentially harmful events are called ‘hazards’ in OHS terminology, and workplace aggression is an obvious and serious hazard that health personnel should not have to accept as a normal part of the job [3, 4]. Identifying the prevalence rates of these hazards, their perpetrators, and the consequences of these hazards for labour supply are important to the individual employee, broader work environment, employer, and health sector in general and are the responsibility of labour and health authorities.

### Sustainable health and welfare services

Sustainable health services require effective and systematic OHS efforts to prevent health problems and other negative consequences of work among employees [5]. This requirement becomes more visible and critical as recruiting and retaining health personnel become more difficult [6]. The current and expected future shortage of nurses and other health personnel may motivate owners and managers of health services to increase their preventive efforts by identifying and managing risks, including the psychosocial and organizational risk factors that make it difficult to retain and recruit health personnel.

### OHS efforts to target psychosocial and organizational hazards

Traditional OHS efforts have been directed towards the prevention of accidents and injuries. As early as 1993, researchers concluded that the strong focus on injuries and safety has led to the neglect of other work-related health problems within the OHS discourse [7]. Psychosocial risks and work-related stress are increasingly recognized as important OHS concerns, at least in Europe [8–10]. However, even certified OHS management systems do not address all types of risks in the workplace; for example, the inadequacy of efforts to address psychosocial and organizational factors has been reported [11, 12]. To improve preventive measures in the workplace, OHS efforts must be directed towards the relevant hazards, including exposure to workplace aggression and other risk factors that reduce the attractiveness of working in health services.

### Consequences of exposure

The consequences of workplace aggression for employees can be severe and long-lasting. Victims of workplace aggression may experience psychological distress, which can adversely affect their overall well-being and quality of life by increasing stress, anxiety, insecurity, fear, emotional exhaustion, depression [13, 14], PTSD and burnout [15, 16]. Such exposure can also change the way employees value their workplace, and feelings of powerlessness and distrust may become dominant [17]. Workplace aggression increases sick leave rates [18] and is costly to organizations in terms of staff turnover and the subsequent recruitment and training of replacement staff [19, 20].

### Intention to leave

Workplace withdrawal behaviour in the form of sick leave, intention to leave the job, or spending less time working depends on the labour market conditions and individual employee’s preference for the job and work tasks [21]. With the current and future expected shortage of nurses, it is crucial to understand more about the reasons why nurses do not want to work in health services.

### Perpetrators of physical violence, threats of violence, harassment, and bullying

A meta-analysis revealed that 24.4% of healthcare workers had experienced physical violence in the past year, 33.2% had experienced threats of violence, and 12.4% had experienced sexual harassment. The highest prevalence rates are in Asia and North America, and as suggested by the authors, more information about the perpetrators is needed to be able to prevent such exposure [22].

### Theoretical underpinnings – investment theory

The financial burden of psychosocial workplace aggression is substantial both to the exposed individual and to society [23]. For the workplace, when an employee quits, whether voluntarily or involuntarily, costs accrue in the form of replacement costs, such as training costs and loss of productivity [24]. If exposure to violence, harassment, or bullying increases intention to leave, improving nurse retention by reducing the risk of exposure can be viewed as an investment decision.

In a simplified model in which the main issue is high turnover rates because of exposure to workplace aggression, the owner of the workplace (public or private) will choose to invest if the cost of the investment is lower than the monetary value of the reduced turnover. When turnover becomes more costly because of a shortage of nurses, the investment in preventive measures becomes more profitable. However, to calculate the profitability of such investment, more information about the costs of not preventing workplace aggression must be obtained; that is, the prevalence of such exposure and the effects of preventive measures must be known. Investment in the work environment and improved OHS practices to reduce workplace aggression are most profitable if they reduce sick leave and turnover rates given that these contribute to the highest costs and, thus, to the greatest gains in an investment analysis.

It is doubtful whether all such exposures can be prevented, and all risks eliminated. However, handling the exposure in a way that does not lead to employees wanting to leave the workplace will help in the retention of nurses despite the risk of exposure. That is, the negative consequences can be mitigated by a sufficient organizational response. If the status quo costs of turnover related to workplace aggression are higher than the investment in effective prevention measures, health services should invest in preventive programs that reduce workplace aggression and associated costs.

### This study

The first objective of this study was to assess the 12-month self-reported prevalence rates of exposure to physical violence, threats of violence, sexual harassment, and bullying among RNs in Norway. The second objective was to identify whether the perpetrators of violence and harassment were colleagues, managers, subordinates, or patients and their relatives. The third objective was to investigate whether the probability of wanting to quit the job (intention to leave) is related to exposure to workplace aggression during the past 12 months. The final objective was to frame workplace aggression from an occupational health and safety perspective.

## Methods

### Study design

To calculate prevalence rates, a cross-sectional design was chosen because we wanted to know how many RNs are exposed to such hazards over a given time interval, in this study, within the past 12 months.

### Setting

Norway has a semi-decentralised health system, with four regional health authorities that are responsible for specialist care, while the municipalities are responsible for primary care and social services [25]. The Norwegian labour market is characterized by a low and stable unemployment rate and a strong tradition of cooperation between employer organizations, labour unions, and the government [26]. Employers have a duty to ensure that the working environment and level of safety are appropriate and satisfactory. The Norwegian Labour Inspection Authority supervises workplaces to ensure that they comply with the requirements of the Working Environment Act [27]. By law, it is a requirement that workplaces and employers ensure a systematic, well-documented, and targeted approach to OHS activities at the workplace; for health service managers, this includes an obligation to identify and assess OSH hazards [28].

Requirements regarding the psychosocial working environment in the Norwegian Working Environment Act include the following: (1) The work shall be arranged to preserve the employees’ integrity and dignity; (2) Efforts shall be made to arrange the work to enable contact and communication about the undertaking with other employees; (3) Employees shall not be subjected to harassment or other improper conduct; and (4) Employees shall, as far as possible, be protected against violence, threats, and undesirable strain as a result of contact with other persons (Sections 4–3).

### Sample

#### Recruitment of participants

In September 2021, a national survey was conducted among RNs in Norway. A total of 30,070 RNs who responded to a national survey about the COVID-19 situation in September 2020 [29] received an invitation to participate in this study. The email addresses from the member register were used to distribute personal web links to the survey. In total, 13,045 (43.4%) responded. However, 1,664 (5.5%) did not meet the inclusion criterion (currently employed in health or care services) and were excluded. Of the remaining 11,381 nurses, 8,800 (77.3%) responded to a translated and validated version [30] of the Copenhagen Psychosocial Questionnaire (COPSOQ III) [31]. These respondents comprised the sample analysed in this study and represented 29.3% of those initially invited to participate in the study.

#### Measurements

##### Background variables

Gender, age, and type of health service the RNs worked in were included as background variables. Nineteen different health services were included, and these cover both primary and specialist health services.

##### Exposure to workplace aggression

Exposure to workplace aggression was assessed with the following four questions from the COPSOQ III questionnaire: Have you been exposed to physical violence at your workplace during the last 12 months? Have you been exposed to threats of violence at your workplace during the last 12 months? Have you been exposed to undesired sexual attention at your workplace during the last 12 months? Have you been exposed to bullying at your workplace during the last 12 months? The following text was included before the question about bullying: ‘Bullying means that a person repeatedly is exposed to unpleasant or degrading treatment and that the person finds it difficult to defend himself or herself against it.’ The response options were ‘No’, ‘Yes, daily’, ‘Yes, weekly’, ‘Yes, monthly’, and ‘Yes, a few times’. For binary regression analysis, these were coded 0 for ‘No’ and 1 for all other answers. For ordinal analysis, these were coded as 0 for ‘No’, 1 for ‘Yes, a few times’, 2 for ‘Yes, monthly’, 3 for ‘Yes, weekly’, and 4 for ‘Yes, daily’.

All four questions about exposure to workplace aggression were followed by ‘If yes, from whom?’, and the respondent could indicate more than one perpetrator. The options were ‘Colleagues’, ‘Manager/superior’, ‘Subordinates’, and ‘Patients/service users/relatives’.

Multi-hazard exposure refers to exposure to multiple hazards [32].

##### Intention to leave/turnover intention

Self-reported turnover intention is the strongest predictor of actual turnover [33, 34]. A single item from the ‘Commitment to the Workplace’ dimension in the COPSOQ III [31] was used to identify the intention to leave: ‘How often do you consider looking for work elsewhere?’ The response options were ‘Always’, ‘Often’, ‘Sometimes’, ‘Seldom’, and ‘Never/almost never’. In the binary analyses, this variable was coded 1 for ‘Always’ or ‘Often’ and 0 for other answers. In the ordinal analyses, this was coded as 1 for ‘Never/almost never’, 2 for ‘Seldom’, 3 for ‘Sometimes’, 4 for ‘Often’, and 5 for ‘Always’ so that a higher value indicated a stronger intention to leave the workplace.

### Statistical analysis

We first present the descriptive results, such as prevalence rates and conditional prevalence rates, according to the type of exposure, i.e., multi-hazard exposure. We next present the results of the binary logistic regression analyses to identify the differences in the odds of exposure to the different types of workplace aggression between RNs across gender, age, and type of health service. We then present the findings for the role of the perpetrator or offender according to the four types of hazards. After presenting the descriptive statistics for the intention to leave according to the background variables and exposure, we present the odds ratios (OR) for the current intention to leave and exposure according to the four types of workplace aggression analysed using separate regression models, both with and without background variables.

To test the robustness of the results, we conducted ordinal logistic regression analyses to determine whether the results were vulnerable to the simplifications in the exposure variables and in the intention to leave variable, and we include these results in the Supplementary results.

All analyses were performed using Stata/SE 16.1 for Windows (64-bit x86-64).

## Results

### The sample

The national sample of 8,800 RNs in Norway was sufficiently representative of the entire population of RNs in terms of gender and geography. However, the youngest age group was less representative of the Norwegian population of RNs than the older, more experienced age group (see graphical illustration in Supplementary Figure S1).

### Prevalence rates of exposure to violence, harassment, and bullying

The 12-month prevalence rates for exposure were 17.0% (n = 1,494) for physical violence, 32.5% (n = 2,863) for threats of violence, 12.6% (n = 1,108) for sexual harassment, and 10.5% (n = 992) for bullying (Table 1). Exposure to threats of violence occurred most frequently; 0.8% reported that they experience this daily and 3.4% weekly.

**Table 1:** Prevalence of exposure to workplace hazards, n (%)

	No	Yes, a few times	Yes, monthly	Yes, weekly	Yes, daily	Yes, total
Physical violence	7,306 (83.0)	1,253 (14.2)	137 (1.6)	86 (1.0)	18 (0.2)	1,494 (17.0)
Threats of violence	5,937 (67.5)	2,047 (23.3)	441 (5.0)	302 (3.4)	73 (0.8)	2,863 (32.5)
Sexual harassment	7,692 (87.4)	952 (10.8)	95 (1.1)	48 (0.5)	13 (0.1)	1,108 (12.6)
Bullying	7,878 (89.5)	777 (8.8)	88 (1.0)	45 (0.5)	12 (0.1)	922 (10.5)
Exposed to ≥1 hazard		3,384 (38.5)	653 (7.4)	395 (4.5)	96 (1.1)	3,749 (42.6)

In total, 42.6% of the RNs had experienced at least one type of such exposure during the past 12 months. Most of the exposed RNs reported that it had happened a few times during the past 12 months; 38.5% reported that one or more of these types of exposure occurred a few times, and 1.1% reported daily exposure to one or more of these hazards.

### Multi-hazard exposure

**Fig. 1:**
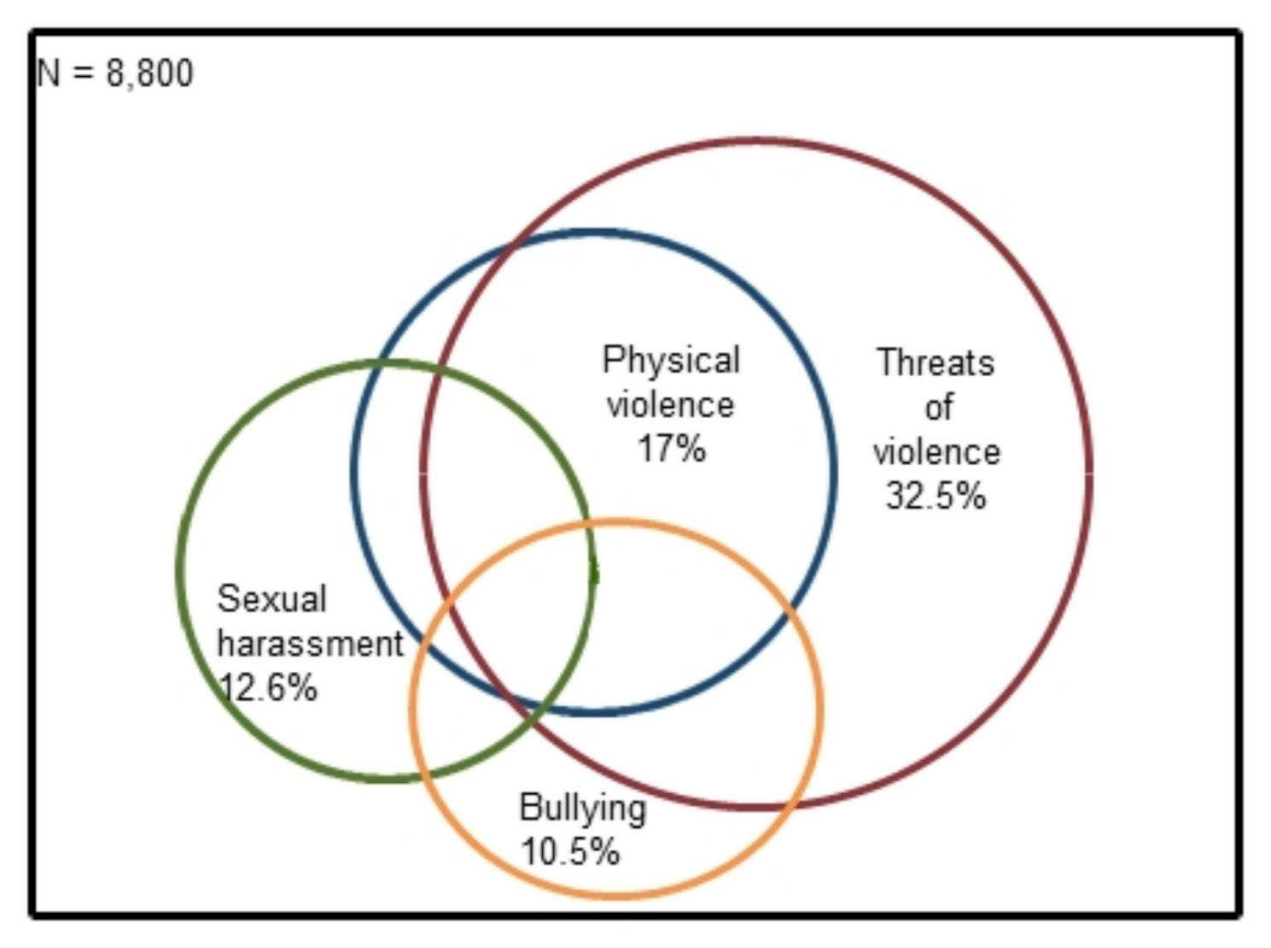
Multi-hazard proportional Venn diagram, modified to include the four hazard variables

Multi-hazard refers to exposure to multiple hazards [32]. As shown by the overlaps in the circles in the adjusted proportional Venn diagram in Fig. 1, exposure to these hazards commonly co-occurred within a 12-month period.

Most of the RNs who were exposed to physical violence were also exposed to threats of violence (88.3%), and almost half (46.1%) of those exposed to threats of violence were also exposed to actual physical violence (Table 2). Among those exposed to sexual harassment, 44% were also exposed to physical violence and 69% to threats of violence. Among those who reported that they were exposed to bullying, 27.8% reported being exposed to physical violence, almost half (48.9%) to threats of violence, and 24.2% to sexual harassment.

**Table 2:** Exposure to different hazards in the context of exposure to other hazards, n (%)

	Physical violence	Threats of violence	Sexual harassment	Bullying
Physical violence		1319 (88.3)	488 (32.7)	256 (17.1)
Threats of violence	1319 (46.1)		765 (26.7)	451 (15.8)
Sexual harassment	488 (44.0)	765 (69.0)		223 (20.1)
Bullying	256 (27.8)	451 (48.9)	223 (24.2)	

### Exposure according to gender, age, and type of health service

**Fig. 2:**
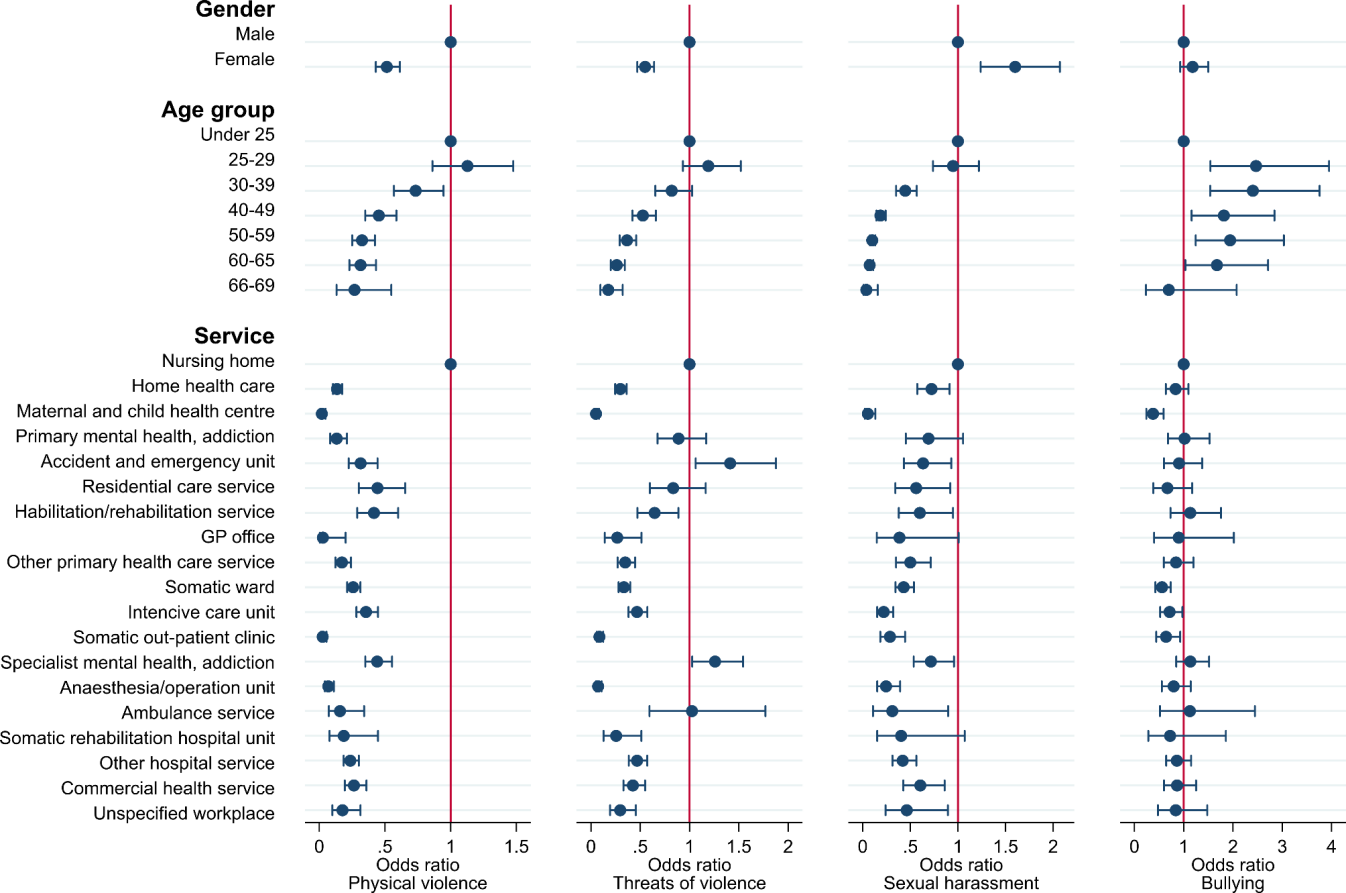
Odds ratios and 95% confidence intervals from the binary logistic regression analyses. The dependent variables are exposure to the four different types of workplace aggression

Compared with male RNs, female RNs had significantly lower odds of being exposed to physical violence and threats of physical violence and significantly higher odds of being exposed to sexual harassment (see Fig. 2). The odds of being bullied did not differ according to gender. Older RNs had lower odds of being exposed to physical violence, threats of physical violence, and sexual harassment than younger RNs. In contrast, the odds of being bullied were highest in RNs in the age group of 25–60 years compared with the youngest and oldest age groups. RNs working in nursing homes had significantly higher odds of being exposed to physical violence and sexual harassment. RNs working in accident and emergency units had the highest odds of being exposed to threats of violence, and those working in maternal and child health centres had the lowest odds.

### Perpetrators of workplace aggression

**Fig. 3:**
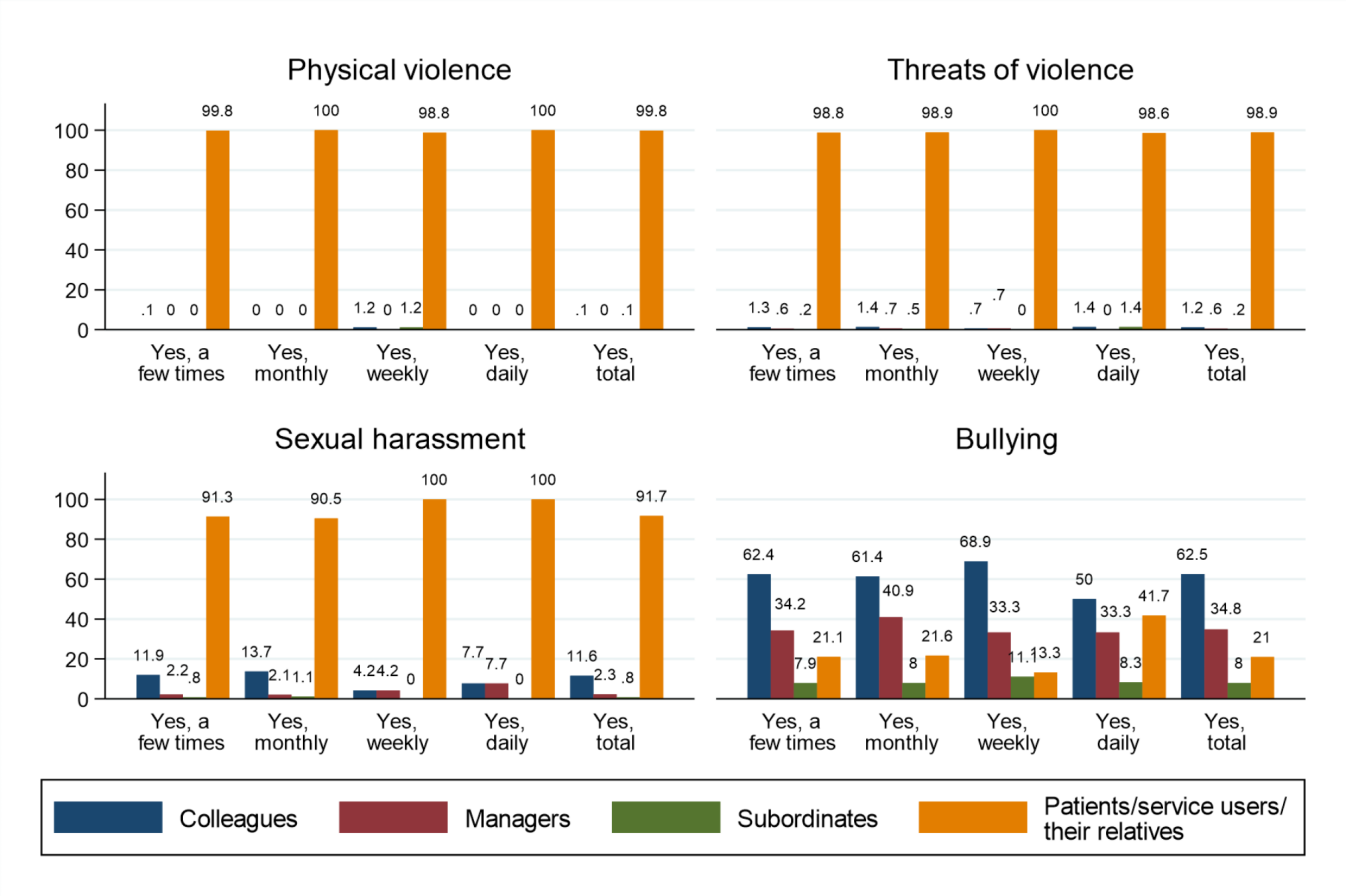
Frequency of exposure to types of workplace aggression according to the role of the offender and exposure frequency

Physical violence and threats of physical violence are almost exclusively perpetrated by those the RNs provide services to, that is, patients or health service users or their relatives (Fig. 3). All RNs who reported daily or weekly exposure to sexual harassment stated that the perpetrators were patients or their relatives. Of those who reported daily exposure to such behaviour, 7.7% stated that the perpetrator was a colleague, and the same percentage reported that the perpetrator was a manager/superior. Of those who reported daily exposure to bullying, 41.7% reported that patients/service users or their relatives were the offender.

In the analysis that did not differentiate according to the frequency of exposure (Yes, total in Fig. 3), patients/service users or their relatives were reported as the perpetrators for 99.8% of physical violence, 98.9% of threats of violence, 91.7% of sexual harassment, and 21% of bullying. The perpetrators of bullying were most often colleagues (62.5%), followed by managers (34.8%) and subordinates (8%). The perpetrators of sexual harassment were reported as colleagues (11.6%), managers (2.3%) and subordinates (0.8%).

### Turnover intention

As seen from the first row of Supplement Tables 1 and 23.6% of RNs never or almost never considered looking for work elsewhere, while 22.5% seldom considered looking for work elsewhere. Those with the highest odds of having intention to leave were assumed to be the 20.6% and 5.4% who replied that they often or always, respectively, considered looking for work elsewhere.

**Fig. 4:**
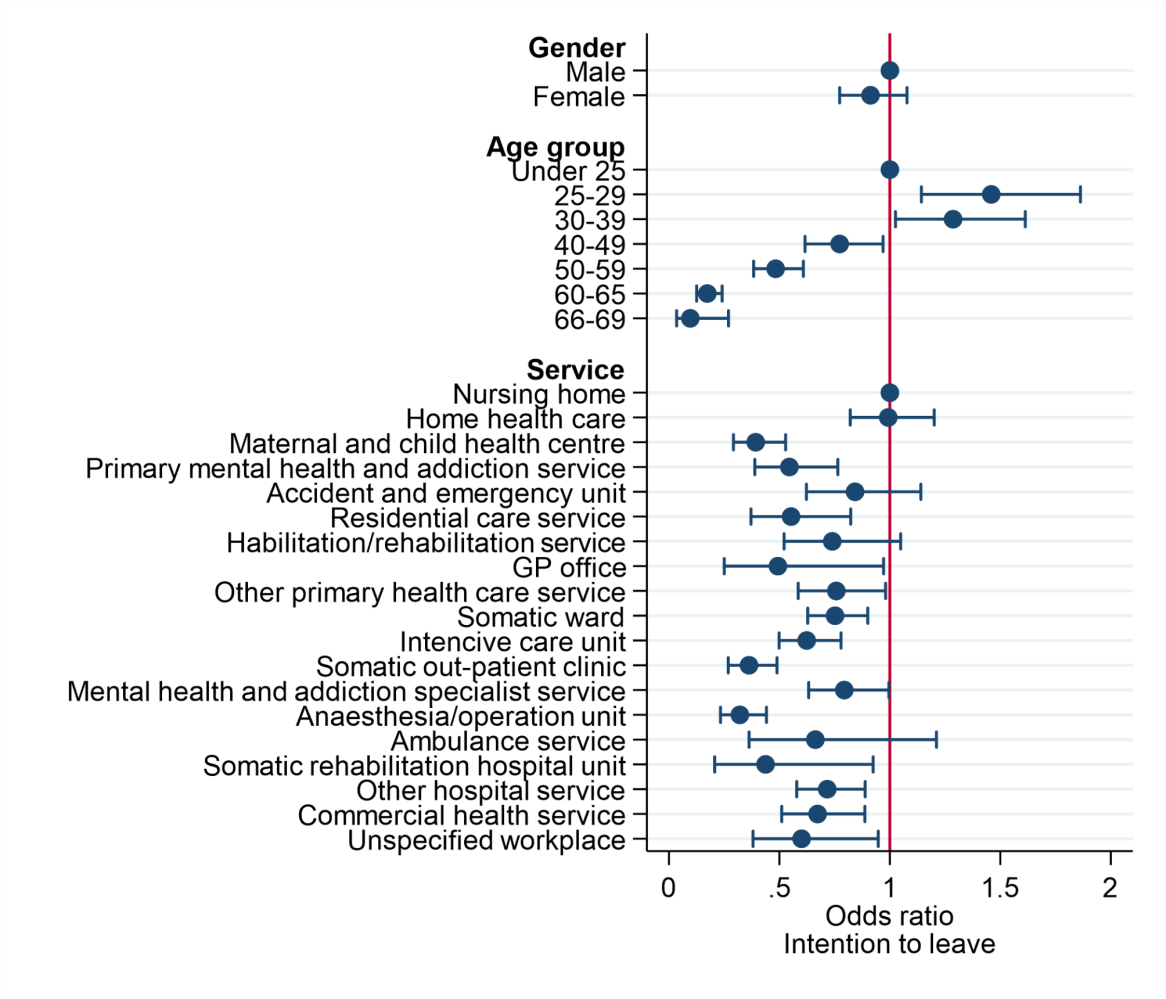
Odds ratios of the intention to leave (always of often looking for work elsewhere) from binary logistic regression analysis

The gender difference in the frequency of looking for work elsewhere (turnover intention) was small and not significant; however, the turnover intention varied significantly between age groups, with the highest odds in the age group of 25–39 years and the lowest odds in the oldest age group (see Fig. 4). The highest odds of turnover intention were found in nursing homes and in home care services. However, this did not differ significantly from the frequencies reported by RNs working in accident and emergency units and ambulance services given the wide confidence intervals relating to the large variability in intention to leave in these services when controlling for gender and age group.

The lowest odds of turnover intention are found in anaesthesia/operation units and somatic outpatient clinics in hospitals and in maternal and child health centres in primary health care services.

### Exposure and intention to leave

We now turn to the analyses of intention to leave and exposure to workplace aggression. As seen in Table 1, few RNs were exposed to different hazards daily. Therefore, weekly and daily exposures were combined into one category in the regression analyses.

**Fig. 5:**
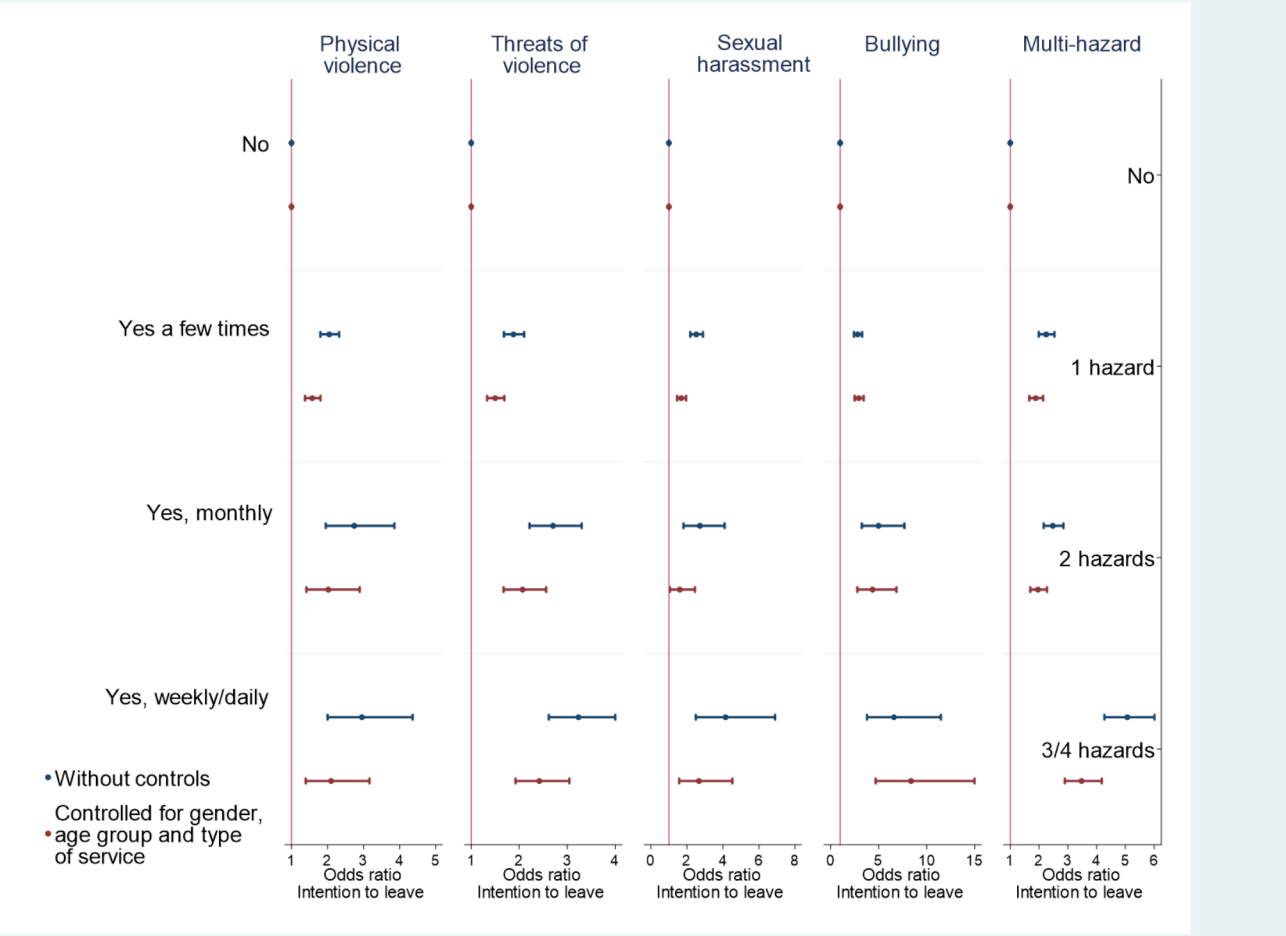
Odds ratios and 95% confidence intervals from the binary logistic regression analyses. The dependent variable was intention to leave the workplace, with and without controlling for gender, age group, and type of health service

Exposure to all types of workplace aggression significantly increased the odds of turnover intention among RNs (see Fig. 5). The largest effect sizes were found for bullying, and such exposure increased the odds of wanting to leave the job the most. Controlling for gender, age group, and type of services reduced the odds of wanting to leave in all cases except for those weekly or daily exposed to bullying, where the odds increased when controlling for gender, age and type of services.

Being exposed to physical violence increased the odds of having intention to leave by 1.58 to 2.95, depending on the frequency of the exposure and whether control variables were included (see Table 3). Exposure to the threat of violence increased the odds of having intention to leave by 1.50–3.23, to sexual harassment by 1.61–4.15 and to bullying by 2.82–8.38.

**Table 3:** Odds ratios (OR) and 95% confidence intervals from binary logistic regression analyses. The dependent variable was intention to leave the workplace, with and without controlling for gender, age group, and type of service

	Physical violence		Threats of violence		Sexual harassment		Bullying	
	OR	[95% Conf. Interval]	OR	[95% Conf. Interval]	OR	[95% Conf. Interval]	OR	[95% Conf. Interval]
Without controls:								
Yes, a few times	2.05	[1.804–2.323]	1.88	[1.682–2.102]	2.52	[2.191–2.891]	2.82	[2.429–3.284]
Yes, monthly	2.74	[1.95–3.855]	2.70	[2.214–3.299]	2.72	[1.811–4.092]	5.00	[3.249–7.680]
Yes, weekly/daily	2.95	[2.003–4.359]	3.23	[2.614–3.998]	4.15	[2.495–6.891]	6.60	[3.795–11.473]
With controls:								
Yes, a few times	1.58	[1.375–1.805]	1.50	[1.331–1.691]	1.69	[1.454–1.956]	2.94	[2.501–3.445]
Yes, monthly	2.02	[1.414–2.894]	2.07	[1.674–2.559]	1.61	[1.058–2.446]	4.37	[2.784–6.854]
Yes, weekly/daily	2.10	[1.395–3.162]	2.42	[1.923–3.044]	2.66	[1.569–4.521]	8.38	[4.69–14.975]

### Robustness analyses

We simplified the analyses of the odds of being exposed to different hazards by generating a binary variable (not exposed and exposed). In the results shown in Fig. 2, we did not differentiate between the frequencies of daily, weekly, monthly, and a few times. To test the robustness of this simplification, we used an ordered logistic model that included all information from the exposure variables, i.e., the dose or frequency of exposure. As shown in Supplementary Figure S2, the results did not differ much from those of the original analysis because there were few observations in the categories other than ‘No’ and ‘Yes, a few times’ (as shown in Table 1).

The same simplification was used in the analysis of intention to leave in Fig. 4. As shown in Supplementary Figure S3, using all information, that is, all five categories in the ordered turnover intention variable (ordinal logistic regression), did not change the results to a large degree.

## Discussion

### Summary of results

The 12-month prevalence rates among RNs showed that 43% had experienced at least one type of workplace aggression. One-third had experienced threats of violence, and almost 1/5 had been exposed to physical violence, while sexual harassment and workplace bullying were reported by 13 and 11%, respectively. Male RNs were more likely to be exposed to physical violence and threats of violence, whereas female RNs were more exposed to sexual harassment. No gender differences were found in the odds of being exposed to bullying. Older age reduced the odds of being exposed to physical violence, threats of physical violence, and sexual harassment, and the youngest and oldest RNs were less likely to be exposed to bullying than those in the middle of the age distribution.

Working in nursing homes or in mental health and addiction units increased the odds of being exposed to physical violence. Threats of physical violence were most frequently experienced in RNs working in accident and emergency departments and mental health and addiction services, whereas sexual harassment was most common in nursing homes, home care services, and mental health and addiction services. Bullying was less common in maternal and childcare services and in somatic wards than in other health services.

Most of the perpetrators who committed physical violence, threats of violence, and sexual harassment were patients/service users or their relatives. Bullying was most frequently committed by colleagues and managers/superior.

We found a clear and strong statistical dependence between exposure to all four types of workplace aggression and odds of RNs’ intention to leave their job. The odds of turnover intention were almost double among those exposed to physical violence, threats of physical violence or sexual harassment compared with those not exposed to these forms of aggression. However, the strongest association was for bullying, which multiplied the odds of looking for work elsewhere between 3 and 8.

Although the working conditions in highly developed countries are good compared to those found in many developing countries, there are challenges in the working environment in countries with long traditions for strong industrial relations and protection of employees in the form of strict requirements for the working environment [35]. The results presented here support this.

### Studies about workplace aggression among nurses

Workplace aggression is much studied in emergency medical services [36–40] and in mental health services [41–43]. Less is known about exposure to this behaviour in home care services and in other primary health services [44]. Our results imply that exposure to workplace aggression is prevalent in health services in general, and measures to prevent such exposure should be taken. More knowledge is needed about effective organisational interventions to prevent or reduce violence directed towards healthcare workers [45]; however, multicomponent interventions seem to be needed [46, 47].

### Prevalence in Norway compared with studies in other countries

A systematic review and meta-analysis showed that one in five health-care professionals experience workplace physical violence perpetrated by patients or visitors worldwide annually [48], which corresponds to the prevalence revealed in our study of RNs in Norway.

We found that RNs working in nursing homes, home care services, and mental health and addiction services were especially at risk of workplace aggression. A previous study of the prevalence of occupational violence in health personnel employed in primary out-of-hours care from Norway found that 78% had been verbally abused, 44% had been exposed to threats, 13% had been physically abused, and 9% had been sexually harassed during the past 12 months [49]. That study also reported that the influence of drugs and mental illness were the most frequently perceived causes for episodes of physical abuse, threats, and verbal abuse.

A systematic review and meta-analysis involving 331,544 health-care workers found that 1/4 reported exposure to physical violence in the past year, 1/3 were exposed to threats, and approximately 12% were exposed to sexual harassment [22]. This analysis found wide variation between countries, study locations, practice settings, work schedules, and occupations. The sample included all healthcare workers and not only nurses, and presented the average prevalence rates of many studies. We found a slightly lower prevalence rate of physical violence but similar prevalence rates of threats of violence and sexual harassment. In our study, 11% of the RNs reported exposure to bullying during the past 12 months; this frequency was lower than the rate of 20–25% of nursing staff who experienced bullying behaviour reported in a review of the literature [50]. Our findings suggest that RNs in Norway have an average exposure to threats of violence and sexual harassment and a slightly lower risk of being exposed to physical violence and bullying than the average nurse included in previous studies.

### Patients as perpetrators of workplace aggression

As stated in the introduction, workplace aggression is defined as behaviour that is intended to harm or intimidate employees [1]. Physical violence and threats of physical violence are almost exclusively carried out by those the RNs provide the services to (patients/service users or their relatives). It is difficult to know the intention of patients and service users; they might not have intended to harm employees. This uncertainty might contribute to underreporting such exposure.

A recent systematic review of nurses’ rationale for underreporting patient- and visitor-perpetrated workplace violence found that the most important nursing factors included nurses’ fear of consequences after reporting, their perceptions, and their lack of knowledge about the reporting process [51]. Common management factors that contributed to the underreporting included a lack of visible changes after reporting, a non-supportive culture in which to report, and the lack of penalties for perpetrators. Furthermore, organizational factors such as the lack of policies/procedures/training, as well as a lack of an efficient and user-friendly reporting system, were found to be important [51].

Previous studies have noted that many nurses consider violence by patients as an inherent part of a nurse’s job [4]. It can be taboo for care personnel to talk about patients as perpetrators because such behaviour may be caused by the patient’s illness or personal crisis [52]. That some patients ‘cannot help it’ is often used as an excuse for such incidents, and both managers and staff may choose to overlook aggression by patients because they know that the behaviour is not personally related to the healthcare worker [53]. However, pretending to be immune to physical and verbal aggression instead of doing everything possible to prevent such incidents may be a poor strategy [54, 55]. Instead, initiating preventive measures that could reduce violent behaviour among patients and that integrate them into the systematic OHS effort by identifying triggers and developing strategies and procedures to handle aggressive behaviour, for example, among older people living with dementia in nursing homes, seems to be a better strategy [56].

### Bullying

The results of our study indicate that those exposed to bullying have a much higher odds of wanting to leave their job than those exposed to aggressive behaviour by patients. Another study found that exposure to bullying behaviour is associated with more severe health-related outcomes for RNs than aggression by patients or their next of kin [57]. However, in line with our results, harassment perpetrated by patients/relatives has been found to have a greater effect on job burnout than harassment initiated by managers or colleagues, which instead causes turnover intention among hospital nurses [58]. A systematic review that aimed to review the literature on workplace bullying among nurses and identify characteristics of anti-bullying interventions concluded that anti-bullying interventions were effective and that upgraded intervention strategies reflecting the contemporary nursing context are needed to ensure workplace bullying prevention [59].

### Multi-hazard exposure

We find that multiple and overlapping hazards are common; however, this is less researched [32], and more research is needed to understand the mechanisms behind this finding. Theoretically, there could be two main explanations for multi-hazard exposure: the individual characteristics of the victims or that some workplaces have an accumulation of hazards because the patients or service users are more likely to expose nurses to different types of aggression. The first would postulate that some nurses are more likely to experience such behaviour because of their individual characteristics such as personality, behaviour, past experiences, or ability to reduce aggression before the situation escalates. However, our results indicate that multi-hazard is a workplace problem because there are large differences in prevalence rates between different types of health services.

### Systematically higher exposure among young nurses

In our study, young RNs reported systematically higher exposure to physical violence, threats of violence, and sexual harassment than more experienced RNs. Other studies have also found that novice nurses are more exposed to workplace aggression [60, 61]. These findings could suggest a higher occurrence of workplace aggression in workplaces where young nurses typically start their careers. However, the age difference remained significant after controlling for the type of workplace. This association may reflect selection effects if the more experienced RNs had left aggressive workplaces when they had the opportunity. The systematic age difference could also suggest that more experienced RNs have different expectations or a generally higher tolerance for workplace aggression and a higher threshold for defining an episode as such and thus systematically report lower exposure rates. Another possibility is that managers or more experienced RNs delegate work tasks that carry a higher risk of workplace aggression to younger RNs, possibly with the intention of increasing tolerance among younger nurses or reducing their own workload and exposure [62–64].

### Working environment act

As stated in the Working Environment Act (Sects. 4 − 3 point 4), employees shall, as much as possible, be protected against violence, threats, and undesirable strain because of contact with other persons. ‘Other persons’ includes patients and their relatives, and the question is whether health services employers do as much as possible to prevent workplace aggression. The high prevalence rates found in this study suggest that the prevention effort in health services in Norway should be improved.

### Broadening the preventive perspective

A qualitative study of healthcare workers employed in emergency departments showed that staff shortages, lack of financial resources, and a high workload are the most common barriers to implementing violence prevention measures [65]. Hence, some workplaces may need to improve workplace culture and balance the workload to avoid high exposure for some healthcare workers. Much could probably be learned from the prevention measures taken in emergency, mental health, and addiction departments to reduce aggressive behaviour by patients. However, effective prevention guidelines must also be tailored to the needs of primary care and, especially, nursing homes and home care services, where most RNs are exposed to aggression.

A broader perspective of OHS primary prevention, including organizational and physical measures, is needed. This may include increased staffing, routine moving of violent patients to a sheltered ward with higher staffing, innovations using specific methods, such as music and art, to reduce aggressive behaviour, virtual reality technology to train RNs and other health personnel in curbing violent situations, and physical activity and individually tailored outdoor activities to calm patients with aggressive behaviour.

RNs’ tolerance for workplace aggression by their patients is probably too high, and preventive efforts are probably too weak. Although unpredictable situations will always occur, knowledge and competence must be used to promote protection and to support RNs in dealing with workplace aggression of all forms. To retain RNs within health care, workplace aggression and other social and organizational hazards must be prevented using all available measures, and these efforts must be integrated into OHS efforts to achieve a dynamic and systematic approach.

Others have argued that healthcare employers should provide better support services when healthcare professionals are assaulted, that the legal system must acknowledge that assaults against nurses are a violation of human rights and that violence should not be tolerated as part of working in mental healthcare settings [66].

### Investing in preventive measures to improve nurses’ working conditions

Each alternative risk-reduction policy should be evaluated against a wide spectrum of criteria, including turnover costs, which will increase in the coming years because of nurse scarcity in many countries, including Norway.

In recent years, managers have begun to include employee health and safety alongside traditional priorities [67]. When deciding whether to invest in the work environment, both the costs and potential benefits from improving the work environment must be considered. Although the direct monetary costs of investing may be evaluated easily, it is more complicated to detect the costs of not investing and the benefits from investing.

### Strengths and weaknesses

Studying a topic that increases the respondents’ interest in participation can produce a skewed sample and excessively high prevalence rates. This study was not presented in the context of workplace aggression mapping, and it is unlikely that the study would have attracted a disproportionate number of RNs who have been exposed to workplace aggression. The large sample size allowed us to analyse differences in exposure and turnover intention across 19 different types of health services; to our knowledge, this has not been done before. The sample bias towards more tenured RNs indicates that the calculated prevalence rates may be too low because young RNs experienced greater exposure to workplace aggression than did more experienced RNs.

### Further research

As noted by others, there is a need for research on the implementation of tools for screening for violent behaviour to shift the focus from managing to preventing violence [65] and workplace bullying [68]. We also need more knowledge about organisational interventions for preventing and minimising workplace aggression. The multi-hazard issue also needs more research. Because 46% of the RNs exposed to threats of violence were also exposed to actual physical violence, such threats should be taken seriously to prevent dangerous situations in health services. Of the RNs exposed to sexual harassment, 69% were also exposed to threats of violence and 44% to actual physical violence. Finally, the question of whether interactions between hazards or differing patterns of hazards amplify the risk needs more research. Studies with multilevel longitudinal designs where workplaces and individuals are followed over time are needed.

## Conclusions

Managers of health services should upgrade their OHS understanding and efforts to prevent exposure to workplace aggression to retain health personnel and to secure the skill supply given that high turnover rates are a major challenge in health services. The results indicate a clear need for improvements in the psychosocial working environment for RNs in Norway. To contribute to providing sustainable health services, labour and health authorities should join forces to develop effective workplace measures to strengthen prevention, mitigation, and preparedness regarding incidents of workplace aggression and response and recovery regarding such incidents that could not be prevented.

### Electronic supplementary material

Below is the link to the electronic supplementary material.

Supplementary Material 1

## Data Availability

The datasets used and/or analysed during the current study are available from the corresponding author upon reasonable request.
