# Supplementary material for: Registered nurses’ exposure to workplace aggression in Norway: 12-month prevalence rates, perpetrators, and current turnover intention
Source: BMC Health Serv Res. 2023 Nov 16;23:1272. doi: 10.1186/s12913-023-10306-z (PMC10655393; doi:10.1186/s12913-023-10306-z)
Supplement: Supplementary file 1 — Supplementary Material 1 [file 12913_2023_10306_MOESM1_ESM.docx]

# Supplementary results

**Fig. S1** Graphical illustration of representativity of the sample compared to the population of RNs from the member register of Norwegian Nurses Association

**Table S1** Descriptive statistics grouped according to the frequency of considering looking for work elsewhere (intention to leave), *N* = 8,769

|  | Never/almost never | Seldom | Sometimes | Often | Always | % of the sample | Chi-squared (df) |
| --- | --- | --- | --- | --- | --- | --- | --- |
| Total | 2,066 (23.6) | 1,969 (22.5) | 2,460 (28.1) | 1,804 (20.6) | 470 (5.4) | 100 |  |
| Gender |  |  |  |  |  |  | χ^2^ (1) = 3.04 *p* = 0.0811 |
| Male | 202 (22.7) | 185 (20.8) | 255 (28.7) | 177 (19.9) | 69 (7.8) | 10.1 |  |
| Female | 1,864 (23.7) | 1,784 (22.6) | 2,205 (28) | 1627 (20.6) | 401 (5.1) | 89.9 |  |
| Age group, years |  |  |  |  |  |  | χ^2^ (6) = 1,005.2 *p* < 0.001 |
| <25 | 88 (20.7) | 76 (17.8) | 115 (27) | 115 (27) | 32 (7.5) | 4.9 |  |
| 25–29 | 105 (12.5) | 142 (17) | 235 (28.1) | 263 (31.4) | 92 (11) | 9.5 |  |
| 30–39 | 258 (15) | 326 (18.9) | 481 (27.9) | 493 (28.6) | 164 (9.5) | 19.6 |  |
| 40–49 | 414 (18) | 540 (23.5) | 744 (32.4) | 481 (21) | 116 (5.1) | 26.2 |  |
| 50–59 | 665 (26.8) | 637 (25.6) | 735 (29.6) | 393 (15.8) | 55 (2.2) | 28.3 |  |
| 60–65 | 469 (51.3) | 235 (25.7) | 145 (15.8) | 55 (6) | 11 (1.2) | 10.4 |  |
| 66–69 | 67 (75.3) | 13 (14.6) | 5 (5.6) | 4 (4.5) | 0 (0) | 1.0 |  |
| Type of service |  |  |  |  |  |  | χ^2^ (18) = 312.8 *p* < 0.001 |
| Nursing home | 187 (16.8) | 214 (19.3) | 341 (30.7) | 271 (24.4) | 97 (8.7) | 12.7 |  |
| Home health care | 172 (18.7) | 179 (19.4) | 251 (27.2) | 254 (27.5) | 66 (7.2) | 10.5 |  |
| Maternal and child health centre | 118 (25.9) | 130 (28.5) | 142 (31.1) | 58 (12.7) | 8 (1.8) | 5.2 |  |
| Primary mental health and addiction service | 66 (24.4) | 72 (26.6) | 82 (30.3) | 40 (14.8) | 11 (4.1) | 3.1 |  |
| Accident and emergency unit | 55 (21.6) | 51 (20) | 70 (27.5) | 62 (24.3) | 17 (6.7) | 2.9 |  |
| Residential care service | 36 (21.4) | 32 (19) | 64 (38.1) | 29 (17.3) | 7 (4.2) | 1.9 |  |
| Habilitation/rehabilitation service | 46 (23.4) | 34 (17.3) | 65 (33) | 43 (21.8) | 9 (4.6) | 2.2 |  |
| General practitioner office | 20 (32.8) | 14 (23) | 16 (26.2) | 7 (11.5) | 4 (6.6) | 0.7 |  |
| Other primary health-care service | 126 (28.1) | 92 (20.5) | 123 (27.4) | 89 (19.8) | 19 (4.2) | 5.1 |  |
| Somatic ward | 219 (17.3) | 266 (21) | 376 (29.6) | 317 (25) | 91 (7.2) | 14.5 |  |
| Intensive care unit | 157 (22.8) | 160 (23.2) | 210 (30.5) | 137 (19.9) | 25 (3.6) | 7.9 |  |
| Somatic outpatient clinic | 167 (35.8) | 128 (27.4) | 109 (23.3) | 56 (12) | 7 (1.5) | 5.3 |  |
| Mental health and addiction specialist services | 171 (26.7) | 142 (22.2) | 162 (25.3) | 135 (21.1) | 31 (4.8) | 7.3 |  |
| Anaesthesia/surgical unit | 138 (32.3) | 119 (27.9) | 116 (27.2) | 44 (10.3) | 10 (2.3) | 4.9 |  |
| Ambulance service | 13 (21.7) | 12 (20) | 19 (31.7) | 9 (15) | 7 (11.7) | 0.7 |  |
| Somatic rehabilitation hospital unit | 16 (31.4) | 14 (27.5) | 12 (23.5) | 6 (11.8) | 3 (5.9) | 0.6 |  |
| Other hospital service | 190 (25.3) | 189 (25.1) | 184 (24.5) | 154 (20.5) | 35 (4.7) | 8.6 |  |
| Commercial health service | 118 (31.1) | 85 (22.4) | 86 (22.7) | 72 (19) | 18 (4.7) | 4.3 |  |
| Unspecified workplace | 51 (35.2) | 36 (24.8) | 32 (22.1) | 21 (14.5) | 5 (3.4) | 1.7 |  |
| Exposed to physical violence |  |  |  |  |  |  | χ^2^ (4) = 211.99 *p* < .001 |
| No | 1,834 (25.2) | 1,716 (23.6) | 2,042 (28.1) | 1,372 (18.9) | 314 (4.3) | 83.0 |  |
| Yes, a few times | 201 (16.1) | 217 (17.4) | 355 (28.4) | 358 (28.6) | 119 (9.5) | 14.3 |  |
| Yes, monthly | 17 (12.4) | 21 (15.3) | 37 (27) | 39 (28.5) | 23 (16.8) | 1.6 |  |
| Yes, weekly | 13 (15.1) | 15 (17.4) | 21 (24.4) | 26 (30.2) | 11 (12.8) | 1.0 |  |
| Yes, daily | 1 (5.6) | 0 (0) | 5 (27.8) | 9 (50) | 3 (16.7) | 0.2 |  |
| Exposed to threats of violence |  |  |  |  |  |  | χ^2^ (4) = 378.26 *p* < 0.001 |
| No | 1,629 (27.6) | 1,434 (24.3) | 1,610 (27.2) | 1,028 (17.4) | 210 (3.6) | 67.4 |  |
| Yes, a few times | 331 (16.2) | 402 (19.7) | 630 (30.9) | 520 (25.5) | 159 (7.8) | 23.3 |  |
| Yes, monthly | 64 (14.5) | 73 (16.6) | 120 (27.2) | 134 (30.4) | 50 (11.3) | 5.0 |  |
| Yes, weekly | 34 (11.3) | 49 (16.2) | 88 (29.1) | 94 (31.1) | 37 (12.3) | 3.4 |  |
| Yes, daily | 8 (11) | 11 (15.1) | 12 (16.4) | 28 (38.4) | 14 (19.2) | 0.8 |  |
| Exposed to sexual harassment |  |  |  |  |  |  | χ^2^ (4) = 226.9 *p* < 0.001 |
| No | 1,920 (25.1) | 1,777 (23.2) | 2,180 (28.5) | 1,439 (18.8) | 346 (4.5) | 87.4 |  |
| Yes, a few times | 129 (13.6) | 156 (16.4) | 254 (26.7) | 308 (32.4) | 104 (10.9) | 10.8 |  |
| Yes, monthly | 8 (8.4) | 26 (27.4) | 18 (18.9) | 34 (35.8) | 9 (9.5) | 1.1 |  |
| Yes, weekly | 8 (16.7) | 9 (18.8) | 7 (14.6) | 15 (31.3) | 9 (18.8) | 0.5 |  |
| Yes, daily | 1 (7.7) | 1 (7.7) | 1 (7.7) | 8 (61.5) | 2 (15.4) | 0.1 |  |
| Exposed to bullying |  |  |  |  |  |  | χ^2^ (4) = 353.73 *p* < 0.001 |
| No | 1,977 (25.2) | 1,847 (23.5) | 2,200 (28) | 1,479 (18.8) | 347 (4.4) | 89.5 |  |
| Yes, a few times | 80 (10.3) | 109 (14.1) | 228 (29.5) | 266 (34.4) | 91 (11.8) | 8.8 |  |
| Yes, monthly | 5 (5.7) | 9 (10.2) | 21 (23.9) | 38 (43.2) | 15 (17) | 1.0 |  |
| Yes, weekly | 3 (6.7) | 3 (6.7) | 11 (24.4) | 16 (35.6) | 12 (26.7) | 0.5 |  |
| Yes, daily | 1 (8.3) | 1 (8.3) | 0 (0) | 5 (41.7) | 5 (41.7) | 0.1 |  |

**Fig. S2** Odds Ratios and 95% confidence intervals from the binary and logistic regression analyses of exposure to workplace aggression.

**Fig. S3** Odds Ratios and 95% confidence intervals from the binary and ordered logistic regression analyses of turnover intention.
